# Creating Political Will for Action on Health Equity: Practical Lessons for Public Health Policy Actors

**DOI:** 10.34172/ijhpm.2020.233

**Published:** 2020-12-05

**Authors:** Fran Baum, Belinda Townsend, Matt Fisher, Kathryn Browne-Yung, Toby Freeman, Anna Ziersch, Patrick Harris, Sharon Friel

**Affiliations:** ^1^Southgate Institute for Health, Society & Equity, College of Medicine and Public Health, Flinders University, Adelaide, SA, Australia.; ^2^Menzies Centre for Health Policy, School of Public Health, The University of Sydney, Sydney, NSW, Australia.; ^3^Menzies Centre for Health Governance, School of Regulation and Global Governance, College of Asia and the Pacific, Australian National University, Canberra, ACT, Australia.

**Keywords:** Political Will, Social Determinants of Health, Health Equity, Health Policy, Advocacy, Australia

## Abstract

**Background:** Despite growing evidence on the social determinants of health and health equity, political action has not been commensurate. Little is known about how political will operates to enact pro-equity policies or not. This paper examines how political will for pro-health equity policies is created through analysis of public policy in multiple sectors.

**Methods:** Eight case studies were undertaken of Australian policies where action was either taken or proposed on health equity or where the policy seemed contrary to such action. Telephone or face-to-face interviews were conducted with 192 state and non-state participants. Analysis of the cases was done through thematic analysis and triangulated with document analysis.

**Results:** Our case studies covered: trade agreements, primary healthcare (PHC), work conditions, digital access, urban planning, social welfare and Indigenous health. The extent of political will for pro-equity policies depended on the strength of path dependency, electoral concerns, political philosophy, the strength of economic and biomedical framings, whether elite interests were threatened and the success or otherwise of civil society lobbying.

**Conclusion:** Public health policy actors may create political will through: determining how path dependency that exacerbates health inequities can be broken, working with sympathetic political forces committed to fairness; framing policy options in a way that makes them more likely to be adopted, outlining factors to consider in challenging the interests of elites, and considering the extent to which civil society will work in favour of equitable policies. A shift in norms is required to stress equity and the right to health.

## Background

**Key Messages**

** Implications for policy makers**

- The paper explains why, despite evidence, including that from the World Health Organization (WHO) Commission on the Social Determinants of Health (CSDH), political will for action is often lacking.
- The paper provides evidence from eight detailed policy case studies of the factors that work for or against action to reduce health inequities by addressing the social determinants of health inequities.
- There is often little political will for action to reduce health inequities. This paper will help policy-makers who desire to reduce health inequities understand the ways in which political will can be mobilized.

** Implications for the public**

There has been a global trend towards increasing health inequities which in turn reduces overall population health status and so affects everyone in the population. Political will to take action on the social determinants of these inequities through improving factors such as income and wealth distribution, housing, education, quality employment and access to health and social services is often lacking. This paper examined eight case studies of policy action outside the health sector to determine what factors influence whether political will for action will exist. The paper then proposes how public health policy actors can create political will through: determining how path dependency that exacerbates health inequities can be broken, through working with sympathetic political forces who are committed to fairness; framing policy options in a way that makes them more likely to be adopted, and outlining factors to consider in challenging the interests of elites and the extent to which civil society will work in favour of equitable policies. Using these strategies to encourage health promotion and health equity policies will improve health for all.

Despite growing evidence and calls for action on the social determinants of health equity since the World Health Organization (WHO) Commission on the Social Determinants of Health (CSDH)^1^ and a series of Lancet Commissions,^2-5^ political action has not been commensurate. The result has been increased rather than decreased health inequities^6-8^ and the social inequities^9,10^ that underpin them. The lack of action can be attributed, arguably, to lack of political will^11-14^ because, however strong the evidence, unless those who hold political power are willing to implement action through policy measures, then progress towards achieving health equity between and within countries will be limited. An increasing number of commentators point to the importance of the political determinants of health and have called for more attention to these.^11-16^ Yet despite its central importance to public health little is known about how political will operates to successfully enact pro-equity policies or not. We start by defining our understanding of the concepts of health equity and political will which are central to this paper.

 Health inequities have been defined by Whitehead^17^ as those “differences in health which are not only unnecessary and avoidable but, in addition, are considered unfair and unjust.” Braveman and Gruskin^18^ extend this definition and see that equity in health occurs when there are no systematic inequalities in the distribution of health because of unfair distribution of resources or other unjust or unfair processes (such as discrimination). The CSDH used this definition and developed a framework to explain how health inequities develop. The framework contains three parts: (1) socioeconomic and political context; (2) socioeconomic position; and (3) intermediary determinants of health. This paper is concerned with the political context and the ways in which it affects health equity through the public policy cycle of agenda setting, formulation and implementation.

 Political will is most simply defined as “the extent of committed support among key decision-makers for a particular policy solution to a particular problem.”^19^ In addition, context shapes political will by influencing leaders’ perceptions and behavior,^20^ which in turn are shaped by formal and informal rules of institutions – the framework within which leaders choose their course of action. Brinkerhoff ^21^ notes that such frameworks incorporate: (*a*) individual actors along with their aspirations, motivations and capacities; (*b*) organizations within which individuals function and on whose behalf individuals often act; (c) socio-economic and governance systems which affect both constraints and incentives for *individuals* and organizations; and (*d*) the public policies, programmes and activities that actors and organizations identify with or are involved in delivering. These mirror the actors’ power, ideas, political contexts and issue characteristics identified as crucial to generating political priority for action on health issues eg, maternal mortality, obesity prevention.^15,22^ While some literature considers political will as a central issue, relatively little empirical analyses exist devoted to unpacking the factors that generate and sustain political will for health equity. Here we address this gap and unpack these factors.

 Research has conceptualised a range of factors influencing political will in regard to social determinants of health equity (summarized in Box 1).^16,23-28^

**Box 1. What Existing Research Says About Creating Political Will for Action on Social Determinants of Health Equity**

** Facilitators **

- Left wing ideology stressing state intervention for the benefit and well-being of the population
- Political feasibility (which happens when windows of opportunity open as problem, political, and policy streams converge)
- Political leaders endorse social perspective on health
- Legal frameworks to support policy action
- Supportive laws which assert human rights
- Cohesive civil society movements eg, the People’s Health Movement

** Constraints**

- Right wing ideology stressing individual free choice
- Strength of medical and behaviourist model overwhelms arguments for social model of health
- Path dependency supporting a curative or lifestyle focus in health sector
- Bounded rationality (decision-making based on limited information processing capability)
- Fiscal constraints that impede new policy directions
- Long time-lag to see change from action on Social Determinants of Health
- Resistance from interest groups

Strand and Fosse^29^ in Norway, Collin and Hayes^30^ in Canada, and Baum et al^25^ in an Australian study found that left wing political ideology is generally more aligned with universal policies, while a right wing ideology emphasizes targeted interventions and is less sympathetic to health equity. Right wing ideology is most typically deeply rooted in individualism holding people rather than their social and economic circumstances responsible for their health and so seeing health inequities as resulting from behaviours and thus less amendable to public policy change.^31,32^ Political feasibility was also important in these three studies. Kingdon^33^ explains that feasibility happens when the three streams of problem, policy and politics merge to open a window of opportunity. However real action to address health equity often requires challenging the status quo and this may create perceived risks and potential insecurity for politicians that make action less feasible.

 A further crucial issue that has been highlighted by political scientists in terms of winning political will is the way that issues are framed. Knight^34^ argues that reframing could be important in gaining political will for seemingly intractable problems. Finnemore and Sikkink^35^ see that policy actors use frames strategically to bring attention to a particular issue and persuade others of its importance. If they are successful, their framing ‘resonates with public understandings, and is adopted as new ways of talking about and understanding issues’ (p. 897).

 Path dependency in policy, the tendency of institutions to retain policy directions and preferences rather than change or reform them, has also been identified as a potentially hindering factor given there has been little existing support for health equity as a policy goal in most countries.^36,37^ Cairney and St Denny^38^ examine why prevention is hard to get on policy agenda and conclude that governments have rarely turned the rhetoric of ‘prevention is better than cure’ in to a set of detailed, consistent, and defendable policies. Oliver^39^ notes that bounded rationality, fragmented political institutions, resistance from concentrated interests, and fiscal constraints usually lead political leaders to favour incremental policy changes rather than comprehensive reforms even when faced with serious public health problems like increasing health inequities. He notes that disrupting problematic path dependency requires a political movement that will change public opinion and voting patterns. He also argues that politicians find it easier to act in support of ‘deserving’ rather than ‘undeserving’ groups – policies in favour of health equity can easily be construed as being about the undeserving poor.

 Policies with the greatest potential to reduce inequities, such as income and wealth redistribution, generate the least political will as they threaten those benefiting from the status quo. Action exists for the least influential policies, such as expanding clinical healthcare services.^40-43^ Short electoral cycles reduce incentives for politicians to support progressive social policies to address health inequities.^44,45^ Laws such as those mandating human rights encourage political will.^46^

 We address the question of how the political will for pro-health equity policies can be created by analysing eight policy case studies and refine key lessons for policy actors.

## Methods

 This analysis is part of the work program of the Australian National Health and Medical Research Council Centre for Research Excellence in the social determinants of health equity.^47^ It is based on qualitative case study methodology, which has been used previously to explain factors affecting the Australian policy agenda^48^ and is suited to examining a contemporary social phenomena in its real-world setting.^49^ Eight case studies were undertaken of Australian policies where action was either taken or proposed on health equity or where the policy seemed contrary to such action (see Box 2).^47^ We selected policy cases that appeared unsupportive or supportive of health equity in order to highlight the differences between these and so gain the benefits of studying multiple cases as recommended by Stake.^50^ This was an important aspect of our study design as negative cases can be as revealing as positive cases. We also sought policies in sectors that are commonly associated with the promotion of health equity and those that are not because of the considerable literature arguing for intersectoral action to address health inequities (see for example Leppo et al^51^). Such policy areas also often have great potential to improve population health. Telephone or face-to-face interviews were conducted with 192 state and non-state participants associated with the case studies (see Table 1 for details).

**Box 2. Policy Case Study Description**

** Paid Parental Leave**

In 2009 the Australian government committed to a limited national PPL scheme. The policy allows for 18 weeks’ pay at the minimum wage for the primary caregiver. Three policy goals were enshrined in the legislation: increased women’s workforce participation, greater gender equality, and improved maternal and child health. Evaluations of the scheme have demonstrated improvements for health equity, especially for women who are low paid or on insecure and contingent employment contracts.^52,53^

** The Northern Territory Emergency Response**

An intervention by the Federal Government in 2007 in response to a report on child sexual abuse in Aboriginal communities in the Northern Territory^54^ involved an army presence in remote communities and bans on alcohol and pornography proclaimed in public notices, seen as shaming by the communities. The NTER originally also included compulsory child sexual abuse checks, although these were subsequently dropped as a result of lobbying.

** The Trans-Pacific Partnership Agreement**

In October 2008 Australia joined negotiations for the TPP, a regional trade agreement with several Pacific Rim countries and led by the United States. Australia signed the TPP in October 2015 (the US subsequently withdrew). We found that the TPP was widely criticized for including provisions that could affect a wide range of determinants of health, including access to medicines, health services, rights for investors to sue governments, and facilitating trade in health-harmful commodities.^55,56^

** Closure of Holden Automotive Manufacturing Plant in Playford, South Australia**

An automotive manufacturing plant in the Adelaide region of Playford closed in 2017 and threatened to increase local unemployment. The policy response did not address health equity and while unemployment was static less secure, casualised part-time jobs increased.^57^

** Australian National Approach to Primary Healthcare**

Australia does not have a PHC policy but there have been a series of initiatives that shape PHC provision including Medicare (the national publicly funded health insurance scheme) that covers fee-for-service General Practice, regional co-ordinating bodies (primary health networks) and Aboriginal community controlled health services.^58^

** Close the Gap**

A bi-partisan national program designed to reduce gaps in health, education and employment outcomes between Indigenous and Non-Indigenous Australians with policy elements that cover health services, employment, education, housing and early child development. The Prime Minister reports progress to Parliament annually.

** Western Sydney City Deal (Urban Development)**

Western Sydney historically contains the most disadvantaged communities in the Sydney region. The WSCD was drawn up in 2018 between local, state and Federal governments. The 20 year deal is a collaboration that leverages off infrastructural investment in Sydney’s second airport to ‘build and coordinate investment that will create world class jobs and quality of life’ in Western Sydney.^59^ Progress on the deal is reported on annually.

** National Broadband Network**

Construction of Australia’s NBN initially designed to provide fibre to the home under the Labor government was downgraded in 2014 to a mix of inferior technologies including re-purposing older infrastructure. Roll out has proceeded slower than originally anticipated, affordability is poor by international standards, and there are considerable inequities in speed and performance of the network.^60,61^

------------------------------

Abbreviations: PPL, paid parental leave; NTER, Northern Territory Emergency Response; TPP, Trans-Pacific Partnership Agreement; PHC, Primary Healthcare; WSCD, Western Sydney City Deal; NBN, National Broadband Network.

**Table 1:** Interviewees in Each Case Study

**Case study**	**Politicians and Advisors**	**Public Servants (Local and State) **	**Civil Society Organisations/trade Unions **	**Industry**	**Academics/Experts/Media**	**Community Members **	**Total **
PPL	5	8	6	4	2	0	25
TPP	5	5	5	5	5	0	25
NTER	5	2	9	0	5	0	21
HC in Playford		10	4	6	0	15	35
PHC Policy	0	13	10	3	4	0	30
CTG	1	13	8	0	0	0	22
WSCD	1	9	2	0	0	0	12
NBN	3	4	8	2	5		22
Total	20	64	52	20	21	15	192

Abbreviations: PPL, paid parental leave; NTER, Northern Territory Emergency Response; TPP, Trans-Pacific Partnership Agreement; HC, Holden factory; PHC, Primary Healthcare; CTG, close the gap; WSCD, Western Sydney City Deal; NBN, National Broadband Network.

 All interview data were thematically analysed using QSR NVivo software and an a priori coding framework drawing on the health equity causal framework identified by the CSDH. Some of the results of this analysis have been used to report on various aspects of our 5 year’s study as reflected in the references in Box 2. Additional analysis was conducted for this paper, which was done through inductive and deductive thematic analysis drawing on the literature on political will summarised in Box 1 to examine these aspects:

- the nature of path dependency;
- whether the issue would impact on staying in or winning government;
- political philosophy (collectivism or individualism), and social construction (positive or negative) of groups affected by the policies;
- presence of economic and/or biomedical framings;
- elite (especially business interests) lobbying;
- civil society and policy advocacy.

This analysis was triangulated by document analysis which consisted of policy documents, media reports, speeches, and enabled examination of each case in order to draw lessons concerning political will and the identification of criteria relevant to shaping political will for health equity. We use these criteria to structure our findings.

 Ethics approval for this research (project 6786) was granted by the Flinders University Social and Behavioural Research Ethics Committee and the free and informed consent of the subjects was obtained.

## Results

 Our case studies included those within the health sector (primary healthcare [PHC]), those related to daily living conditions that matter for health such as work conditions, digital access, urban planning and social welfare (Holden factory [HC], Western Sydney City Deal [WSCD], National Broadband Network [NBN], Paid Parental Leave [PPL], Close the Gap [CTG], NTER [Northern Territory Emergency Response]), and cases that provide insights into global (Trans-Pacific Partnership Agreement [TPP]), national (PHC, NBN) and regional (WSCD, NBN, HC) structural dynamics. The focus also reflected policies at different stages of the policy cycle. Our research found one case was primarily positive for health equity (PPL^52^), four were mixed (PHC,^58^ WSCD, CTG, NBN^61^) and three were negative (NTI,^62^ TPP,^55^ HC^57^).

Table 2 presents the components which indicate the extent to which political will was created for health equity in each case according to the criteria listed above. Each of these elements is discussed below with illustrations from our case studies.

**Table 2:** Political Will in 8 Case Studies of How Policy Addressed Health Equity

**Case Study Description **	**+/- For Health Equity**	**Path Dependency **	**Staying in/Winning Government **	**Political Philosophy: Individualist/Collectivist Construction of Populations Targeted **	**Primary framing: Biomedical/Economic/Social/Behavioural** **/Colonial**	**Interests of Elites **	**Role of Advocacy **
PPL	Positive	First law on topic so broke path dependency	Women’s votes	Collectivist, Universal for women	Gender equality, Economic	Industry successfully argued that payment should be responsibility of government Industry top up voluntary	Crucial influence and advocates were listened to
NTER	Negative	Continued colonial domination, pathologising of Aboriginal people and communities	White Australia votes and appeasing right wing parties	Remote Aboriginal communities, Individualist approach, Targeting Aboriginal men as perceived perpetrators of child abuse	Economic colonial, biomedical	Federal government interest in having power over Northern Territory affairs and Aboriginal communities	Advocates strong but ignored. Some positive impact eg, mandatory sexual abuse checks dropped, and primary healthcare funding was increased
TPP Agreement	Negative	Continued neo-liberal economic focus	Economic growth will win votes	Global neoliberalism, Growing media interest	Economic	Corporate power pro-trade agreements	Advocates vocal, small wins on defense of tobacco control and access to medicines
HC in Playford	Negative	Closure reinforced existing low socio-economic status of area	Provision of jobs and keeping unemployment low will win votes	Removal of subsidies for automotive manufacturing to support free trade, Ex-Holden workers focus rather than whole of deprived community	Economic	State government perceived political risk from rising unemployment and not being seen to act, Disinterest in a lower socio-economic status are with persistent disadvantage	Advocacy weak other than trade unions for rights of workers
PHC Policy	Mixed	Medicare national health insurance scheme continued but still unequal access	Popular appeal of Medicare maintained no co-payments	Collectivist in part, Whole of population but gaps evident in coverage	Primarily biomedical	Medical power maintained general practitioner fee-for-service focus rather than more comprehensive PHC	Medical advocacy strongest, very weak lobby for CPHC except Aboriginal community controlled
CTG	Mixed	Colonialism continued	Bi-partisan support for aspiration and accountability to parliament through annual report on health, education and employment targets	Targeted to Aboriginal and Torres Strait Islanders, Constructed as a problem to be solved; contestation between self-determination and assimilation	Colonial, social behavioural	Top-down political and bureaucratic control of resources	Policy resulted from very strong and well-organised advocacy from multiple groups that understood evidence on social determinants of health and legacy of colonialism but only partial take up by government
WSCD	Mixed	Break with previous planning system and historically limited infrastructural investment in the West of the city (as opposed to the East where the CBD and coast are)	Response to population pressure and growing inequity between west of Sydney and the area nearer CBD. Pressure on traffic, housing prices leading to community dissatisfaction. Increasing number of marginal seats	Collectivist in part, Population of western Sydney to build on ‘strengths’ of the region. Individualist in part because strategy emphasizes entrepreneurialism	Economic, (quality of life links to social)	Airport and globally competitive city region desired by elites Government led strategy with emphasis on global business forces for ongoing delivery leveraging off government investment	Internal government advocacy, limited civil society + social sector engagement
NBN	Mixed	Break with privatization of telecoms to instigate a (temporary) renationalization of infrastructure, Some positives for equity – eg, universal wholesale pricing, government trying to ensure there were cheaper entry level connections, focus on rural and remote in rollout	Originally, delivering effective internet. Became about reducing cost of project to tax payers	Became less collectivist, Whole of population but significant differences in coverage	Primarily economic	Business demand	Consumer and community advocacy on inclusion, rural and remote issues, and affordability, but little effect on policy implementation

Abbreviations: PPL, paid parental leave; NTER, Northern Territory Emergency Response; TPP, Trans-Pacific Partnership Agreement; HC, Holden factory; PHC, Primary Healthcare; CTG, close the gap; WSCD, Western Sydney City Deal; NBN, National Broadband Network; CPHC, Comprehensive Primary Health Care; CBD, Central Business District.

###  Path Dependency 

 Four of our case studies indicated a disruption of path dependency which was positive for health equity. The introduction of Medicare providing for a national publicly funded health insurance scheme (although it was narrowly focused on general medical practice) came with the election of a new Labor government committed to this disruptive move. Subsequently Medicare has become the new norm and hard to disrupt providing an example of path dependency working in favour of health equity. PPL was a national first for Australia, challenging the established, gendered welfare system, and resulted from extensive lobbying by a range of trade union and women’s groups. There was a broad view among the respondents that PPL had been achieved as a result of slow incremental change which broke the previous path dependency.^52^ The WSCD is the first time Federal, state and local governments have collaborated on city planning that includes economic, social and equity aims; responding to rapid growth and increasing geographical inequities within the city. However, the political will for equity behind the policy is currently weak as economic growth and a positive narrative of success dominate over risks or disadvantage. The NBN was designed to break the dominant control of a privatized Telstra.

 In contrast, the NTER continued the sustained long-term colonialization of Aboriginal communities but in the shorter term path dependency was broken in a negative approach to health equity in that the army was brought into remote Aboriginal communities, child health checks were initially proposed to be mandatory, and the Racial Discrimination Act was suspended to facilitate the imposed measures.^63^ One senior Aboriginal doctor noted how a pent up desire for changing the path dependency was seized:

*“There’s a whole lot of things that the Federal government hated about Aboriginal policy and affairs in the Northern Territory and they just wanted to get it all in one fell swoop. And so they threw everything that they didn’t like or were offended by, like closed communities or having to get permits and stuff like that*.”

###  Impact on Staying in or Winning Government 

 The Coalition Government proposed a $7 co-payment under Medicare for visits to a general practitioner in the 2014-2015 budget, but the unpopularity of this with voters meant the proposal was rapidly dropped.^64^ With the announcement of the closure of the HC the State government considered it politically essential to respond with schemes to create new jobs and regenerate the area. There was no perceived incentive however to respond to the continuing disadvantage of the Playford region with specifically pro-equity policies, especially as the electorate was in a safe Labor seat, and the 2017 incoming Liberal government dropped special measures devoted to the region. A local government actor suggested that once the HC was no longer an electoral threat that interest in the region would diminish with this comment:

*“There is the broader context of well now that Holden is on the front page of the paper every day, what is your longer-term policy response, will it just die a natural death because it’s not causing political pain*.”

 Another CTG respondent – a politician – noted that the nature of the political cycle and the desire to be seen to be undertaking new initiatives could also undermine good policy which can address long term disadvantage:

*“They’ve cut money out of – the incoming Liberal cut half a billion - $534m out of the Aboriginal funding bucket, so it’s been a bit of a mishmash and it’s been quite ad hoc. Again, you know, they’ve still maintained this funding approach where it’s ‘we’ll fund that program, then we’ll stop that program. We’ll fund this program’ so it’s – and I suggest that a lot of that is based on the wanting to announce other policy – other programs. It’s the political cycle and a failure, again, to address some of those key underlying issues.*”

 A crucial initiative related directly to the CTG strategy was the Aboriginal proposal for a constitutional voice for Aboriginal and Torres Strait Islander people. Drafted after a long consultative process the Uluru Statement from the Heart^65^ put the case to government for a representative body (recognized in the constitution) which would give Aboriginal and Torres Strait Islanders a say in law and policy affecting them. One Aboriginal respondent noted that the conservative Prime Minister Malcolm Turnbull at the time the Declaration was released was complementary about the declaration yet rejected accepting it. He put this down to internal party politics and the politically unstable governments that has characterized Australian politics since 2007:

*“This reaction feels very much like internal party-political expediency because the excuses given are so not matching the reality; public support seems to be there, the actual issues that were raised about [the Voice being] a third chamber, like a parliamentary chamber doesn’t exist because that’s not what’s being proposed.*”

 A similar point was made in relation to political motives for the WSCD:

*“As an idea, [the WSCD] is a good idea. Obviously there’s political reasons for doing it as well, because Western Sydney is sort of borderline in New South Wales politics. The Liberals got in because they won a lot of Western Sydney seats.*”

 In the case of PPL, advocates secured support from the Australian Labor Party (ALP) prior to the 2007 election and worked to get support for an inquiry into opportunities for PPL as soon as ALP won the election. The legislation required an unusually robust effort by politicians as noted by a public servant:

*“You have to have a champion within government. Somebody who’s really committed to making it happen and I think there was enough of those in the Labor party that pushed it along and made it a priority… they ran around Parliament house getting support, getting the numbers, like they were really good, and you don’t always see that; you don’t often see that actually*.*”*^52^

 The NTER was perceived to be in part an election tactic to be tough on a group who were popularly perceived as underserving recipients of state welfare. A Northern Territory politician noted:

*“It was all about the election, … and I think Alexander Downer [Coalition senior politician] reported that. He’s in the mainstream media saying that.*”

 The WSCD resulted from a political need to be seen to be responsive to the infrastructure needs of a rapidly growing and geographical inequitable region in Sydney. The region is politically contestable, so the desire to retain or gain power also meant political will could be lost in part because political parties wanted to be seen in the electorate as investing in the region. The advantages of being in a region with politically marginal parliamentary seats was recognized:

*“So if you look at City Deals around the country they’ve gone to places where marginal seats decide elections … And you can hear that now, you can hear it in the early rumblings of the State election campaign. You can hear this notion that the West has been getting a raw deal…*”

 This was part of the concern with the NBN where its original universal fibre-to-the premises model was diluted as a result of an election promise which the interviewees saw as political opportunism to generate a point of policy difference during an election campaign. This perspective was summarised by a telecommunications expert as follows:

*“…if Party A says yes then Party B says no, you know, if it’s climate change or NBN or energy or whatever. It’s so partisan, it’s so shocking that [there’s] nothing like a vision or a strategy in any of these fields, healthcare, communications, climate change … [That] can happen if you’ve got this partisan approach to these very important social and economic elements in our society….*”

 A view confirmed by another respondent:

*“I think the real reason was that the Coalition saw political advantage in being seen to be doing something different from what Labor was doing. That’s the way we work in Australia in politics, you wouldn’t dare to be seen doing what your opposition was doing.*”

 The NBN case also highlighted the extent to which internal tensions within parties can impede equitable policy. A senior telecommunications executive noted that a universal fibre to the premises scheme would have been equitable but the policy change to an inferior network reflected such tensions:

*“[The policy change to multi-technology mix] was a purely political decision by a Prime Minister who clearly did not understand the significance of broadband and implemented by a communications minister who was clearly wedged and unable to fight back and so what we saw was the NBN effectively becoming part of a political game or political gamesmanship.*”

###  Political Philosophy 

 The political philosophies adopted by political parties play a crucial role in determining political will for pro-health equity policies. A strongly individualistic perspective leads to policies which predominantly focus on biomedical interventions to treat disease, or behaviourist interventions in relation to smoking, drug and alcohol use, diet and exercise. The two cases in which previous path dependencies were broken in ways favorable to equity – Medicare PPL and NBN were both introduced by centre-left Australian Labor governments. The PPL was a hybrid scheme which continued to allow for additional voluntary contributions from private sector employers so to some extent represented a neo-liberal philosophy, but the result was a universal minimum government funded scheme. A preference for individualism was evident in early responses to PPL when some conservative political actors did not believe the state had the responsibility to provide support for parents. The commitment of the Labor government to an accessible and affordable NBN was shown by this comment from a politician when it was first introduced:

*“I believe strongly … that telecommunications should be considered a utility in the same way as, you know, power, water, postal services … That was at the heart of our thinking. … Utilities are public goods. They should not be privatised and I have fought this my entire political life.*”

 By contrast a more market driven approach was indicated in a Coalitionpolicy statement:

*“But do consumers really need the data transfer rates only fibre can provide, or typically use applications that need such bandwidth? And even if they do, are they willing to pay more for it?*”^66^

 Strong political differences were evident in the PHC case where Labor governments were consistently more committed to the universalist public health scheme and conservative Coalition governments to private health insurance and the introduction of co-payments. There were also significant differences in political acceptance of the evidence on the social determinants of health. One of the respondents in the PHC case noted of the more conservative party:

*“[I]n terms of the health promotion work and preventative health work there is a dislike of certain terms at the political level so things like the social determinants of health is not the sort of - it’s the Voldemort of health discourse in some circles, you know, its name cannot be uttered.*”

 This view was supported by another respondent saying: “*I think part of the issue is that the discussion around social determinants has a political overtone to it which will always alienate the right, basically.*”Respondents also noted the strong commitment to the notion of user pays and private health insurance in the conservative party and attributed this to ideology and noted more commitment to equity in the Labor party. This comment summed up such opinions:

*“Well, the ideology from the current federal government is obviously to spend as little as possible on public health services and make it user-pay. That’s a deeply entrenched belief and whether they do little things to improve access for this or that or the other what they ultimately, deeply desire is a system where primary healthcare is paid for by the person or eventually by private health insurance…*”

 The NTER, designed by the conservative Howard Coalition government, was noticeable for painting a very negative picture of Aboriginal communities and feeding in to existing racist and colonial attitudes. It built on a longer-term view of Aboriginal and Torres Strait Islander people constructed as a “problem to be solved,” also seen in the CTG. This construction of Aboriginal people made it harder for advocates to oppose the draconian move of bringing the army in to those communities and drew on the Australian population’s lack of knowledge and often racist attitudes about remote Aboriginal communities. There was also a clear difference between the two major political parties in response to the CTG strategy. While there was ostensibly bi-partisan support a senior Aboriginal policy actor from an NGO noted:

*“I think once the government changed from the Rudd/Gillard [two Labor Prime Ministers] years into the Abbott/Turnbull [two conservative Prime Ministers] years well, then things really came to a halt and the government wanted to take a different direction. They almost – or not quite but perhaps in practice they did abandon Close the Gap but it seems to have just made a bit of a comeback. I don’t think there’s been the same level of investment.*”

 Part of the reasons for the conservative Coalition’s opposition to PPL concerned the way some politicians regarded the role of women. A public servant reported:

*“There was a strong view amongst a large percentage of the parliament, particularly the Howard parliament that women should not work with children under five*.*”*^52^

 The election of the more socially progressive ALP provided a window for securing the PPL policy.

 While both major political parties in Australia supported a neo-liberal agenda in relation to the TPP there was some difference based on philosophy. This is reflected by the Labor Party’s refusal to include investor state dispute settlement clauses in trade agreements. This move protected the public health regulatory function of government from being challenged for reasons for free trade.

###  Economic and/or Biomedical Framings 

 The trade agreement case provided the most extreme example of economic framing. The TPP was broadly supported by the major political parties in Australia. Civil society respondents noted that a neoliberal market discourse was embedded in institutional processes and structures resulting in market interests being favoured over public interest and public health. A trade union respondent noted:

*“I think that 20 years of neoliberal economics have kind of poisoned a lot of the communication channels, where if you show evidence that the deal won’t increase GDP [gross domestic product] despite what the government says, people find it hard to believe some of that*.*”*^67^

 This market framing was useful for economic arguments for protecting access to generic medicines because this framing aligned with neoliberal principles behind trade liberalization. We only found a few exceptions to the economic framing. The Greens raised issues concerning the equity impact of free trade. A Labor Health Minister was a champion of tobacco control and successive trade ministers supported this position by excluding tobacco control measures from investor state dispute settlement.^67^ The power of the economic globalization to dominate debate was highlighted by the following TPP civil society respondent:

*“….the Labor party fears being painted as being anti-trade and anti- globalisation, so much that it would never think about implementing some of these [progressive health] suggestions.*”

 An emphasis on economic factors was also evident in the HC where the overriding political concern of the State government was to secure jobs for the exiting Holden workers and have a regional economic development plan.^57^ The potential for this latter plan to be equitable was undermined by the failure of the conservative Federal Coalition government to provide funds to rejuvenate the region. The South Australian Labor government’s Our Jobs Plan committed $60 million which was predicated on a commitment from the conservative Federal government to contribute $300 million to help rejuvenate the region. They did not do so which was perceived to have undermined South Australian Labor government’s attempts at alternative strategies for a more equitable regional economic development response. Additionally, the removal of automotive government protections by the Federal government were argued to have been a critical factor in the Abbott Coalition government securing Free Trade Agreements with China, Japan and Korea.^68^ In this case the concern for free trade overrode consideration for the Holden workers. In the HC case local actors were those expressing concern for the wider community and not just securing jobs for the exiting automotive workers as this quote from a local government official demonstrates:

*“So the first group was around state’s government intention to try and get them back into work immediately, so the transition over to work as opposed to being unemployed, and they’d be successful if they achieved that. What hadn’t been looked at necessarily was those wider community impacts to the workers themselves but the wider community around the perceptions, all that kind of stuff. And the fact that getting the job was only one part of the puzzle, because getting a job might have been a job with less money, very different hours, affecting being able to pick up children from school, those kinds of things.*”

 The overriding considerations of the Federal and State conservative governments in the WSCD were economic investment, centred on the new Sydney airport and its potential for job creation, which respondents argued might also be good for equity. However, the economically driven ‘strengths-based’ discourse behind the deal ignores reference to vulnerability or risks to social equity from private sector investment and entrepreneurial based activities.

 In the case of the NBN both major political parties saw that the network would eventually be privatized and neither held a vision for it to be publicly owned as a public good asset. The latter option would have given more scope for equity consideration to be taken into account. Our study indicated that equity, other than a commitment to universal, basic access to some form of telecommunications infrastructure, did not figure in the rollout of the network.^69^

 The CTG strategy did reflect in part a social determinants of health equity framing in that its strategies extended beyond the health sectors and included employment, education and housing. However, much of the framing concerned Aboriginal people living economically productive lives in the terms of mainstream white society. The strategy has done little to challenge systematic racism and the deep institutional roots of colonialism, or to advance self-determination.

 Economic framings can sometimes create political will for pro-health equity policies. Thus, the increasing and projected continuing increasing burden of chronic disease (which disproportionately falls on those lower on the social gradient) meant new models of PHC were being considered and political support for Aboriginal Community Controlled Health Services were growing. However, the regional Primary Health Networks, responsible for planning PHC, were found to have a primary medical focus giving only scant attention to social determinants and health equity.^70^ Economic framings were used strategically to support PPL by advocates developing a framing for economic productivity.^52^

###  Elites’ (Especially Business Interests’) Influence 

 Elite apathy or opposition to an issue has been shown to discourage political will.^71^ Tracking the influence of elites in policy is difficult. They are generally not open to being interviewed and there are few mandates to force disclosure of political influence through lobbying. A coalition of senior elite trade union, politicians, public service, media and industry women threw their weight behind PPL and were important in creating political will for the policy. A community member in the HC case believed that, because the region was a Labor stronghold, conservative political parties would as a consequence not invest in the area:

*“Once you get a change of government, the dynamics move and its lower socio area, it’s a labor area. If a conservative government got in will they care, they’ve never bothered to put money in this area in the past when they’ve been in. in the past, they never put a penny into the area, not a penny.*”

 The TPP analysis indicated that industry actors, through a shared neoliberal framing with government, had more access and influence over the government’s agenda. The TPP illustrated how the global trading system prioritizes economic considerations and supports the interest of business, supported by a network of elite actors arguing the benefits to economic growth of this system.^55^ A public servant recognized this interest:

*“Certainly, the exporters have a very concrete and specific interest. And the agreements are for them. And so you need to ensure that it’s actually going to serve their interests basically*.*”*^52^

 In the case of the PPL advocates worked to secure some industry lobby groups support as a way to overcome entrenched industry opposition to PPL in Australia. In the end industry got what it wanted – a government funded scheme with voluntary contributions from employers and trade unionist noted that “the only way to get it over the line was it if would be taxpayer funded.”^52^

 The colonial mindset evident in the NTER indicated that white elites claiming superiority over Aboriginal culture contributed to the political will for the policy. The analysis of PHC policy indicated a growing business lobby in favour of a privatized health system and suggested the existing bi-partisan support for Medicare was under pressure. In both the NTER and the PHC cases medical power was seen to be powerful in shaping responses. One respondent noted that the Australian Medical Association (AMA) is:

*“…a union for doctors. They have no – apart from Indigenous health – I will give them credit there, but they have shown no inclination over the years to take any consideration of the broader population’s health pretty much at all, other than just general clinical terms.*”

 The AMA as an elite medical group has both good access to and considerable influence over politicians. The powerful influence of medical and industry groups over political will was highlighted by a former very senior public servant when he commented on the range of this influence:

*“I think in healthcare there’s just a dreadful inertia and difficult – and change is difficult because of the power of vested interests and in that I would include private health insurance, the AMA, Medicines Australia and the Pharmacy Guild. I think those four between them effectively control what happens in healthcare in Australia and the ministers see their role, and certainly the Department of Health and Ageing sees its role as to how to manage those vested interests to keep the Minister out of trouble rather than develop healthcare policy.… in the wider field of health promotion the power of the liquor industry, the power of fast foods, are very powerful indeed and they have a significant effect on healthcare in the community and they are having pretty much a free run at the moment.*”

 The power of medicine emerged from our eight cases as one of the strongest factors evident in determining policy. respondent A respondent who was a former senior federal public servant captured this very well:

*“That’s clearly the direction that healthcare reform has to go, it has to go into primary healthcare, but the vested interests don’t want that; it would prejudice their privileged position. As I often say, ministers may be in office but they’re never in power in the health field. The power of vested interests is just too great to get the informed debate and then giving the politicians the courage to make some difficult political decisions based on equity and efficiency.*”

 Thus this form of elite power is able to sap political will for health equity. The WSCD has emphasized private sector investment, principally investment by global firms, to enable the economic ‘strengths’ of the region. Risks to equity with such investment are ignored.

###  Civil Society and Policy Advocates

 Our case studies highlighted many examples where advocacy helped to create political will for health equity. Bi-partisan political support for CTG eventuated after a concerted civil society campaign to ‘Close the Gap.’ Once the policy was adopted the mandated necessity to report to Parliament on progress towards CTG targets has kept the issue in the political spotlight at least annually. PPL was achieved following three decades of lobbying by women’s and trade union groups. Within PHC we found some political support for Aboriginal community-controlled health services which were supported by a strong national advocacy organization and the importance of Medicare was strongly supported by civil society organisations. In the HC the automotive trade union was influential in ensuring the State government focused on supporting the Holden workers to find employment. But there was a lack of commitment to the northern area of Adelaide where the Holden plant was located as noted by this local government official:

*“You know, again, I think there’s just a - there is no focus on Northern Adelaide from the Federal Government in general, and, you know, I would have though, if you’re looking at the city deal type network and a region in change, Northern Adelaide - State Government really have to drive through that, because you need all levels of government. There was a proposal, there’s competing proposals within Adelaide, and the northern one wasn’t successful.*”

 This meant that any lobbying from local government by community groups was unlikely to be effective in face of the lack of commitment from the Federal government to the region. While the focus in the TPP remained on an economic agenda, advocacy by groups concerned about its potential health and welfare impacts gained traction and created some political attention to these alternative agendas.^72^ Advocates are most often thought of as being outside government. Insiders are also important to bringing about political will. Oliver^39^ refers to these government insiders as “investors” who have the position and power in the form of staff, financial resources, authority and personal commitment to help create and support political will. Our interviews found in the PHC that public servants worked to develop and sustain policy support for Aboriginal community-controlled health services, for PPL and to maintain Medicare. It was also noted in the PHC case that public support for Medicare had made it hard for any government to change it:

####  “So, in terms of the political drivers that’s very strong public support for Medicare, so obviously governments have to be very careful if they [ever] change that. So, as we saw at the last election, there’s very strong support for Medicare.”

 Another respondent added to this saying that “*people in Australia clearly care a lot about is having access to affordable healthcare for everybody.” *In a similar veina trade unionist interviewed as part of the TPP remarked that Medicare and pharmaceutical benefits scheme “*were very much like the holy grail. In Australia you can’t touch it.*”^67^ The analysis of the WSCD indicates that it has attracted little civil society engagement and any advocacy for equity came from within government. This respondent from a peak NGO pointed to the token nature of the involvement:

####  “I think at best what may have happened is an invite to a launch of a thing…we were involved with the GSC when it was just a start up…And then what’s flowed on from there we’ve not been involved in. There is a great opportunity to hook the public to the City Deal but they haven’t been drawn into it.”

## Discussion and Conclusion

 Despite widespread recognition of the importance of political will to achieving health equity, little scholarly and advocacy attention has been paid to how to foster it. This may reflect the view of political will as an “idea riddled with ambiguity and imprecision.”^19^ Our empirical research has applied key aspects of political will to an array of policy areas, to give the concept more analytical precision and highlight ways that public health actors can use an understanding of political processes to manufacture, enhance and reinforce political action for health equity. In this discussion we do two things: firstly offer some guidance for public health activists and policy advocates for how they might go about creating political will. Secondly, we detail the changes that are required in political structures and actors for health equity to be advanced.

Box 3 details the areas in which advocates need to consider in order to win political support for health equity. We pose a series of questions designed to help advocates determine their readiness for effective advocacy. These questions cover these areas: determining how path dependency that exacerbates health inequities can be broken, through working with sympathetic political forces who are committed to fairness; framing policy options in a way that makes them more likely to be adopted, and outlining factors to consider in challenging the interests of elites and the extent to which civil society will work in favour of equitable policies.

**Box 3. Creating Political Will: Questions for Public Health Activists and Policy Advocates**

** Path Dependency Assessment **

Is there a strong strand of policy path dependency you will need to challenge to make this policy pro-health equity?

If so what actions/evidence might break this pattern?

Are there windows of opportunity you can use?

** What Are the Implications for Political Parties**

Is this policy area a “hot” political topic?

Will this policy area threaten the incumbent government’s ability to maintain power?

Will it help the opposition gain power?

Is there a chance of bi-partisan support for the policy?

Is there an influential political leader who will champion the cause?

** Collectivist or Individualist Political Philosophy**

To what extent does the policy change require a commitment to a collectivist mindset?

Which political parties express a collectivist ideology?

In parties that do not are there some members who are more sympathetic?

Is there popular will for collectivism in this policy area?

** Primary Framing**

What is the current primary framing in this policy area?

How does this need to change to be more pro-health equity?

Which norms need to become dominant?

Can you make an economic as well as human rights argument for health equity, likely to be influential in era of neo-liberalism?

** Interests of Elites**

Do those who hold elite power support the current primary framing?

How do they work to maintain the framing and their interests?

Are there any weak points in the elite interests’ armour that can be exploited?

What institutions underpin the elite’s power?

How can these institutions be changed?

** Role of Advocacy**

What is the history of civil society action in the policy area?

Which civil society organisations will support the argument for health equity?

Are there allies in public services and within political parties for health equity responsive to civil society pressure?

Advocacy from civil society activists is one important means to win political will. As well as assessing the political scene by asking the questions in Box 3 there are other key issues concerning political will which we now examine. A crucial issue is how political will can be fostered in the key actors, politicians, taking into account that politicians respond to many forces (including evidence, electorates, perception of popular opinion, elite pressure, institutional inertia, political philosophies). While our conclusions are based on case studies from one country we consider that the lessons we have derived will be more widely applicable.

 Greater political will for health equity will require new norms which stress the human right to health and the moral imperative in favour of equity within and between nations. Horton^73^ has noted that the norms that work against human rights are “a rigid consensus among these powerful elites that prevents most attempts to question the norms on which political decisions are made.” Finnemore and Sikkink^35^ propose that norms change through a three-stage process: emergence, creation of a cascade, and internalisation and acceptance. Health equity is at the emergence stage where there is support in civil society and through the Sustainable Development Goals^74^ but there is little normative support when in competition with the profit goal. Our cases illustrated this in relation to the TPP, HC, PPL, the WSCD and a creeping privatization in PHC policy.

 The challenge is to shift the framing of inequities away from a medical and behavioural towards a political framing. The Lancet – University of Oslo Commission on Global Governance noted that the problem of reducing global health inequities can be framed “as a managerial problem, devoid of the conflicting interests and power asymmetries that can distort the underlying mechanisms that determine health inequalities.”^75^ Political will requires a shift to norms which see health inequities as a result of a “toxic combination of poor social policies and programmes, unfair economic arrangements, and bad politics.”^1^ For example, the NTER which was particularly negative for health equity provides a case in which political will underpinned very poor social policy (sending the army in to remote Aboriginal communities to enforce surveillance), unfair economic arrangements (the historic disadvantage of Aboriginal and Torres Strait Islander peoples under colonial Australia) and bad politics (the cynical manipulation of a racist mainstream during a Federal election campaign). The NTER demonstrates how political will is shaped by the social construction of the groups toward whom a public policy is targeted.^24^ The importance of the way in which an issue is framed by lobby and advocacy groups has been recognised by the UK Joseph Rowntree Foundation^76^ and the US Frameworks Institute.^77^ Both organisations work on issue social and eoncomic issues to find ways of changing their framing in order to influence public policy.

 Creating political will to challenge unhealthy policies will require politically-led institutional change to recognize the rights of Indigenous people, acceptance of a benign and partnership approach to social and welfare policy, and a moral shift to adopt a pro-equity position. This position is likely to have broad appeal if backed by strong political leadership and an advocacy movement arguing the moral position for social justice. The CSDH^1^ noted that it had been “impressed by the force of civil society and local movements that both provide immediate local help and push governments to change.” Certainly, the CTG case indicates that civil society advocacy led to bi-partisan support for a pro-health equity policy, making the task of framing and norm shifting much easier and the political will consistent and likely to last across elections.

 In parliamentary democracies short electoral cycles mean that politicians have narrow windows in which to bring about comprehensive changes before they have an eye for the next election. Yet achieving health equity will require politicians to break with many path dependencies and to make some fundamental changes to aspects of practices and institutional ways of working.^24^ The dominance of neo-liberal policies in so many countries^78,79^ mean that a comprehensive policy agenda designed to reduce health inequities would be a radical departure from the policy norms. Such a shift is unlikely to eventuate without strong political leadership to lead and enforce the norm shift. However, in most neo-liberal regimes the scope of debate and policy formulation is narrow because this reduces conflict over core values and beliefs.^39^ Opportunities for large scale innovation only occur infrequently. When such windows of opportunity open health equity policy entrepreneurs and ‘investors’ need to be ready with proposals and fit these to suit the political situation of the time. Without the creation of such political will, health inequities will continue to grow making our world a less healthy and secure place.^80^

## Ethical issues

 Ethics approval for this research (project 6786) was granted by the Flinders University Social and Behavioural Research Ethics Committee.

## Competing interests

 FB, SF, PH, and AZ received a grant from the Australian National Health and Medical Research Council in order to conduct the study. There are no further conflicts of interest to report.

## Authors’ contributions

 Conception and design: FB, MF, PH, AZ, SF; Acquisition of data: FB, MF, BT, PH, KBY, AZ, SF, TF; Analysis and interpretation of data: FB, MF, BT, PH, KBY, AZ, SF, TF; Drafting of the manuscript: FB; Critical revision of the manuscript for important intellectual content: FB, MF, BT, PH, KBY, AZ, SF, TF; Obtaining funding: FB, SF, PH, AZ.

## Disclaimer

 Please note that KBY died on the 19th March but had previously approved the paper.

## Funding

 This research was funded by the Australian National Health & Medical Research Council Centre for Research Excellence Grant APP1078046.
